# From Plastic Pollution to Remediation Solutions: Micro/Nanofiber-Based Strategies for Microplastic and Nanoplastic Removal

**DOI:** 10.3390/membranes16070223

**Published:** 2026-06-29

**Authors:** Dinh Nguyen, Minh-Ky Nguyen, Dinh Duc Nguyen

**Affiliations:** 1 Institute of Research and Development, Duy Tan University, Da Nang 550000, Vietnam; 2 School of Engineering & Technology, Duy Tan University, Da Nang 550000, Vietnam; 3 Faculty of Environment and Natural Resources, Nong Lam University, Hamlet 33, Linh Xuan Ward, Ho Chi Minh City 700000, Vietnam; 4 Department of Civil & Energy Systems Engineering, Kyonggi University, Suwon 16227, Republic of Korea

**Keywords:** micro/nanofibers, plastic pollution, environmental remediation, functional materials, microplastics, nanoplastics, sustainable treatment

## Abstract

The extensive use of plastics in everyday life has exerted a significant influence on the environment, with the release of micro- and nanoplastics posing even greater ecological threats. Plastic contamination, particularly in these smaller forms, has emerged as a pressing environmental concern due to its persistence, bioaccumulation, and potential hazards. Traditional treatment systems are generally ineffective at removing such micro- and nano-scale complex pollutants. Recently, micro- and nanofiber-based materials have emerged as promising candidates due to their large surface area, porous structure, and adjustable functionality, enabling efficient adsorption, filtration, and photocatalytic degradation. The term micro/nanofibers in this study encompasses both electrospun nanofibrous membranes and nanofiber-based functional layers or additives incorporated into pre-existing membrane structures for performance enhancement. The incorporation of photocatalysts enables these materials to promote photocatalytic oxidation, degrading plastics into smaller, less toxic compounds. This paper outlines recent progress in developing micro- and nanofiber systems for environmental remediation, highlighting their design approaches, removal mechanisms, and multifunctional capabilities. Ultimately, the discussion explores emerging directions, existing limitations, and future opportunities, highlighting how these advanced materials can contribute to sustainable and efficient pollution control strategies.

## 1. Introduction

As one of the most critical environmental issues worldwide, plastic pollution results from the continual influx of large quantities of plastic waste into both terrestrial and aquatic environments each year [1,2,3]. The increased consumption of plastic and its effects on humans and aquatic organisms have made micro- and nanoplastic pollution a widely studied global problem. For instance, as plastics fragment into micro- and nanoplastic, fragments persist in soil and water systems, resulting in severe environmental pollution [4,5]. A key issue related to plastic pollution is the presence of plastic debris in aquatic ecosystems. Also, concerns over the ecological and health impacts of micro- and nanoplastics have intensified, given their persistence, ease of transport, and ability to concentrate toxic chemicals. Their persistent and virtually non-biodegradable nature in wastewater contributes to an emerging crisis. It required more consideration to reduce their pollution.

**Figure 1:**
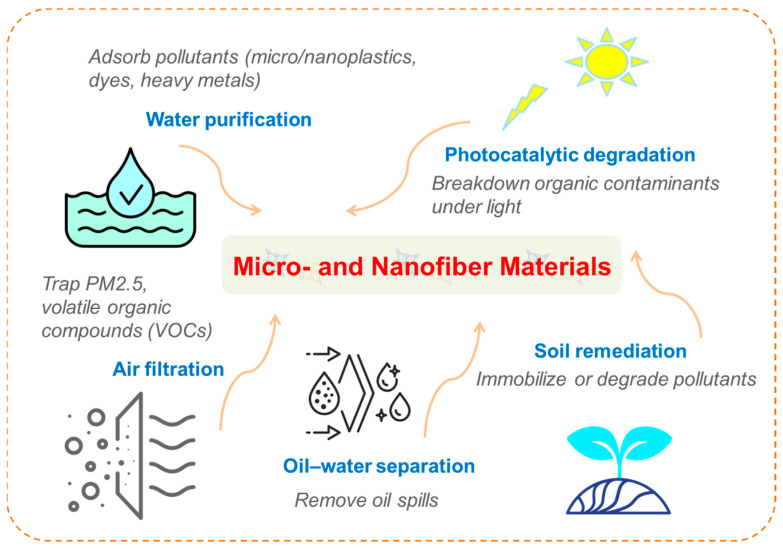
Applications of micro/nanofibers in environmental remediation.

In recent years, micro/nanofiber materials have offered multifunctional capabilities in environmental remediation, including the purification of water, filtration of air, separation of oil and water, photocatalytic breakdown of pollutants, and remediation of contaminated soils [6,7,8,9,10] (Figure 1). The combination of extensive surface area, controllable porosity, and tailored surface chemistry enables micro- and nanofibers to efficiently adsorb or degrade a wide range of contaminants in soil, water, and air. This adaptability underscores their potential for sustainable environmental remediation. While filtration technologies are commonly applied to capture large plastic particles, their efficiency decreases with smaller sizes due to pore blockage in the membrane. Techniques such as primary settling and filter-based systems—including biofilters and ultrafiltration—have been shown to exhibit superior performance in these applications [11]. Membrane bioreactor (MBR) technology is capable of removing microplastics with an efficiency approaching 99%, highlighting its effectiveness in wastewater treatment [12]. Techniques based on membrane separation have gained prominence due to their efficacy in eliminating micro- and nanoscale plastic particles from wastewater streams [13,14,15,16]. Given that membrane materials facilitate pollutant removal via adsorption, electrostatic interactions, and physical capture [17].

Overall, conventional remediation approaches face challenges in efficiently and selectively sustainably treating complex pollutants. Traditional methods are scalable and often achieve removal efficiencies exceeding 90%, whereas hybrid systems can further enhance microplastic removal to beyond 95% [18,19]. Within this framework, micro/nanofiber-based systems have shown considerable potential for environmental remediation, characterized by their well-developed surface area, tunable functionalities, and compatibility with various treatment processes [7,20,21]. Micro/nanofiber-based membranes exhibit very high porosity and specific surface area, forming highly interconnected pore networks that enable significantly enhanced permeability with reduced mass transfer resistance compared with conventional polyamide (PA) and inorganic membranes [6,22,23,24]. Their pore size, fiber diameter, and surface chemistry can be readily tuned, allowing flexible optimization of selectivity, wettability, and antifouling properties that are difficult to achieve with conventional membranes. In addition, micro/nanofiber membranes are generally lightweight, mechanically flexible, and easy to functionalize, facilitating the fabrication of composite or multifunctional membranes. Compared with inorganic membranes, they also offer lower fabrication temperatures and lower costs.

With high surface area, adjustable surface chemistry, and synergistic adsorption–degradation mechanisms, nanomaterials present promising approaches for micro- and nanoplastic removal through adsorption, photocatalysis, and membrane-based filtration [25,26]. Production methods for nanofibers, such as electrospun nanofibers and chitosan-based nanofibrous membranes, have recently been proposed for treating wastewater [7,27,28]. Through recent nanotechnology innovations, a variety of specialized nanomaterials have emerged, providing powerful tools for capturing and decomposing plastic particles [25]. Despite growing interest in micro- and nanofiber-based remediation materials, major gaps remain in understanding the mechanisms governing the capture of microplastics, especially nanoplastics. Moreover, most reported systems are validated mainly at laboratory scale, with limited evidence of long-term stability, fouling resistance, environmental safety, and scalability under realistic water matrices and operational conditions. This review highlights advancements in micro- and nanofiber technologies and their promise to transform plastic pollution into solutions for environmental remediation. It focuses on assessing the efficiency of micro- and nanofiber-based strategies for achieving practical, efficient, and sustainable solutions for the removal of micro- and nanoplastics. In this work, micro/nanofibers are considered in two distinct structural roles within membrane systems. First, electrospun nanofibers can be fabricated directly into self-supporting or composite nanofibrous membranes, where the interconnected fibrous network forms the primary filtration layer. Second, nanofibers may serve as functional modifiers, either embedded within the membrane matrix or deposited as surface coatings onto conventional polymeric or ceramic membranes. In this case, the nanofibers act as performance-enhancing components rather than as the principal structural framework.

## 2. Review Methodology

This study systematically reviews recent advances in microplastic and nanoplastic contamination and highlights emerging micro/nanofiber-based remediation technologies for their removal from aquatic environments. The review covers key aspects including sources, occurrence, environmental fate and transport, ecological and human health risks, and advanced fiber-based mitigation strategies.

A comprehensive literature search was conducted using major scientific databases, including Web of Science, Scopus, and Google Scholar, covering publications from 2020 to 2025 to ensure updated insights. The following keywords and combinations were used: “microplastics,” “MPs,” “nanoplastics,” “NPs,” “sources,” “occurrence,” “fate and transport,” “ecological impacts,” “health risks,” “microfibers,” “nanofibers,” “environmental remediation,” “electrospun membranes,” “adsorption,” “filtration technologies,” and “future perspectives,”.

Additionally, backward and forward citation tracking was performed by examining references cited in the selected articles to identify further relevant studies. Only peer-reviewed journal articles written in English were included to ensure scientific rigor and reliability.

A flow diagram illustrating the literature search and selection process in this field is presented in Figure S1. Initially, a total of 194 records were retrieved based on the predefined search criteria. After eliminating 46 duplicate entries, 148 records remained for screening. Following the title and abstract evaluation, 118 articles were considered for further assessment. Of these, 56 publications, including review papers, were excluded because they did not satisfy the established eligibility criteria. Subsequently, 62 full-text articles were thoroughly evaluated for relevance and methodological rigor. Ultimately, 43 original research articles were retained and incorporated into this comprehensive review.

## 3. Sources, Characteristics, and Adverse Impacts of Plastic Pollutants

### 3.1. Sources and Characteristics

**Figure 2:**
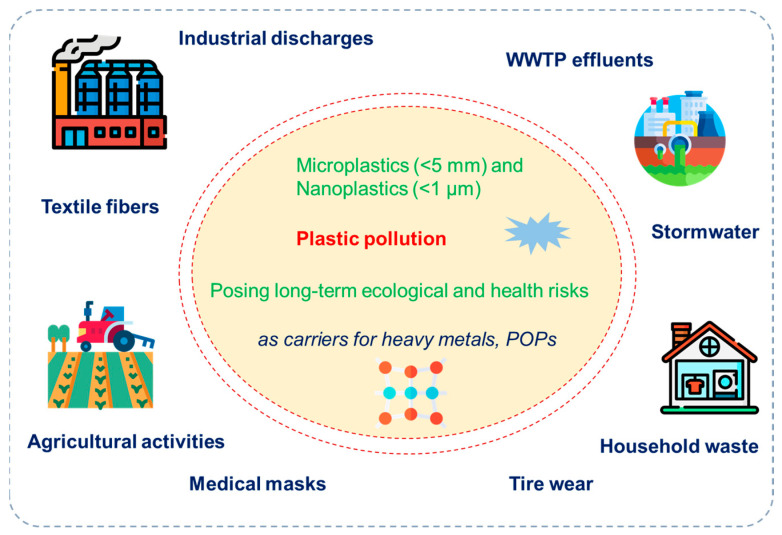
Sources of water contamination and adverse impacts.

Plastic pollution arises from multiple sources, such as industrial discharges, agriculture, wastewater treatment plant (WWTP) effluents, household waste, textiles, tire wear, and the environmental degradation of larger plastics (Figure 2). The widespread production and use of plastics in contemporary industries are dominated by packaging, which is the primary source of plastic waste [29]. Most plastic products are not biodegradable and can persist in the environment for centuries [30]. WWTP effluents represent a major route by which micro- and nanoplastics are released into aquatic environments. Urban expansion and increased plastic use have exacerbated environmental issues, with micro- and nanoplastics increasingly entering water bodies through household, industrial, and stormwater sources, as well as poorly treated wastewater [31,32]. Single-use medical masks made from non-degradable materials raise serious environmental issues and add to plastic pollution [33].

Today, polyolefins, including polyethylene, polypropylene, and polyvinyl chloride, are the predominant petroleum-based plastics, offering strong mechanical properties, low mass, and high stability. Through physical, chemical, and biological mechanisms, these materials slowly fragment into microplastics (<5 mm) and nanoplastics (<1 µm) [10,34]. Characteristics such as size, shape, surface charge, polymer composition, and weathering influence the mobility, longevity, and contaminant interactions of micro- and nanoplastics. Plastic particles are categorized as primary or secondary depending on their source [35]. Primary micro- and nanoplastics are deliberately produced to be small for use in products such as cosmetic cleansers and toothpaste, including microbeads, microfibers, resin pellets, and industrial abrasives [36,37,38,39]. Secondary particles result from the breakdown of larger plastics in the environment through processes such as photodegradation, oxidation, mechanical abrasion, and biodegradation, and represent the primary source of micro- and nanoplastics pollution. Due to their resistance to microbial breakdown, these plastics represent a serious environmental concern. They can spread via air, rivers, and wastewater discharges [40,41].

### 3.2. Adverser Impacts

Micro- and nanoplastics, due to their small size and reactive surfaces, can adsorb toxic substances and bioaccumulate, presenting prolonged ecological and health hazards [42,43]. It is proposed that micro- and nanoplastics carrying micropollutants may enter food webs through digestion by organisms, potentially affecting both ecosystems and human health. Plastic particles can also act as carriers for heavy metals, allowing them to persist longer and spread over wider areas [44]. Plastics incorporate numerous additives, including flame retardants and plasticizers. With increasing micro- and nanoplastics concentrations, these particles can accumulate chemicals from water, thereby elevating their toxicity, environmental impact, and health risks. Their extensive occurrence in natural environments is alarming because they are persistent and can harm both wildlife and humans, entering the food chain via marine organisms [45]. Animals across ecosystems are impacted by micro- and nanoplastics. Plankton and fish consuming these particles may experience blocked intestines, reduced nutrient absorption, immune system disturbances, altered gene expression, and inhibited growth [37,46]. Tiny microplastics (<20 μm) are more abundant in water and exhibit greater biological toxicity [47]. Micro- and nanoplastics pose serious ecological risks. When ingested by aquatic organisms, they disrupt gut colonies and can accumulate through the food chain. In the digestive tract, they may cause abrasion and inflammation in the stomach and intestines [48].

**Figure 3:**
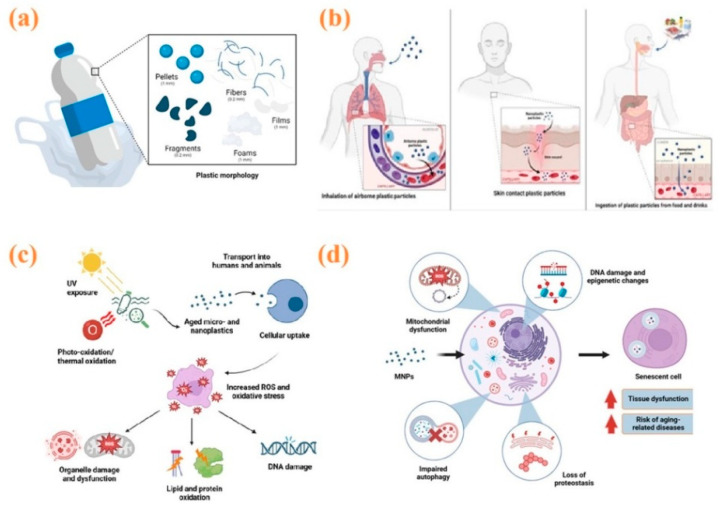
(**a**) Plastic morphology, (**b**) micro- and nanoplastics mainly enter the human body [49], (**c**) micro- and nanoplastic weathering, cellular uptake, and subsequent oxidative stress in cells, and (**d**) micro- and nanoplastics exposure induces molecular hallmarks of aging in human and animal cells [50].

As a result of global plastic pollution, micro- and nanoplastics are present throughout natural environments, especially in water, posing risks to humans and ecosystems. Figure 3 presents the pathways of micro- and nanoplastic exposure and their potential impacts on human health. Researchers have detected plastics in the human body, including in the placenta and blood, indicating a serious health threat [51]. Human exposure to micro- and nanoplastics has been connected to multiple health issues, including toxic effects and an increased risk of cancer [52,53,54].

To sum up, plastic pollutants originate from diverse sources such as packaging waste, synthetic textiles, tire wear, and industrial effluents, and are characterized by their persistence, diverse particle-size spectrum, and strong affinity for co-contaminants. Once fragmented into micro- and nanoplastics, they pose adverse impacts on aquatic ecosystems and human health through bioaccumulation, toxicity, and vector effects. In addition, plastic debris enters oceans via wind, rivers, and currents, harming marine biodiversity and affecting sectors such as tourism and fishing through lost revenue, higher clean-up costs, and a decline in aesthetic appeal [55,56]. These challenges have driven growing interest in micro- and nanofiber materials, whose high surface area, tunable porosity, and surface functionality make them promising platforms for the efficient capture, separation, and remediation of plastic pollutants in environmental systems.

## 4. Micro/Nanofiber Materials and Applications in Environmental Remediation

### 4.1. Overview of Micro/Nanofiber Materials

Micro/nanofiber materials are ultrafine fibers with diameters ranging from the micrometer to nanometer scale, characterized by high surface area, porosity, and tunable chemical functionality [6,8,57]. They can be fabricated using techniques such as electrospinning, phase separation, and self-assembly, allowing precise control over fiber morphology and composition. These materials, classified as polymeric, carbon-based, or hybrid fibers, offer distinct benefits for capturing and breaking down pollutants, depending on their origin and structure. Electrospun nanofibers, integral to these membranes, are strongly influenced by fabrication parameters, including the polymer utilized, voltage, solution feed rate, and the spacing between the needle and collector.

Biopolymer-based nanofibrous materials, characterized by biodegradability, biocompatibility, mechanical flexibility, sustainability, high porosity, and multifunctionality, represent highly attractive platforms for addressing challenges in energy, environmental, and biomedical fields [58]. These materials can be produced using sustainable nanofibers derived from natural biopolymers (such as cellulose, starch, and chitosan) and synthetic biodegradable polymers (including polylactic acid and polycaprolactone).

Predominantly fabricated via electrospinning, nanofibers are commonly derived from polymers such as polyvinylidene fluoride, polyacrylonitrile, and polysulfone, selected for their hydrophilic characteristics and favorable mechanical properties [22,59,60]. Polymer-based membranes, including polyethersulfone, polyethylene, and polypropylene variants, are widely used in the filtration sector due to their mechanical stability, economic viability, and straightforward fabrication processes [61]. In recent years, micro/nanofiber materials have emerged as up-and-coming solutions compared to traditional fibers, owing to their small diameters, adjustable porosity, and lightweight nature. These exceptional properties enable versatile applications in environmental remediation, particularly for treating water, air, and soil.

Carbon nanofibers are among the most promising members of the carbon fiber family [62]. Carbon nanofiber-based nanomaterials have emerged as versatile platforms for energy conversion and storage, catalysis, adsorption/separation, sensing, and biomedical engineering [63]. In particular, three-dimensional (3D) carbon nanofibers structure, featuring high specific surface area, interconnected porous networks, low density, and robust mechanical strength, has garnered growing interest in energy production/storage and environmental science. Various design strategies for producing carbon nanofibers-based 3D nanomaterials, i.e., electrospinning, hydrothermal synthesis, templated synthesis, chemical vapor deposition, and other combined techniques, are summarized with an emphasis on their applications, e.g., water purification and air cleaning.

Recently, nylon-6 micro- and nanofiber composite membranes exhibited a uniform porous structure. For instance, [64] developed nylon-6 micro-nanofiber composite membranes (FCMs) with a 3D uniform gradient pore structure using air-jet spinning with the aid of polyethylene oxide (PEO). The composite membranes demonstrated effective filtration performance toward ultrafine particles. Also, owing to their renewability and biodegradability, polylactic acid (PLA) fiber membranes present clear advantages over conventional materials in water filtration applications. Melt electrospinning has emerged as an efficient and environmentally sustainable method for producing PLA fibers [65]. The multilayer PLA fiber membrane demonstrated excellent water filtration efficiency. Further, TiO_2_ nanofibers have been widely explored for wastewater treatment, with electrospinning serving as an effective approach for producing nanostructured fibrous materials [66]. Such an approach provides a viable and sustainable route toward the development of environmentally friendly and efficient water treatment materials.

In short, micro/nanofiber materials can be fabricated from a wide range of fiber types, including natural polymers, synthetic polymers, carbon-based fibers, and inorganic fibers. Among these, composite fiber materials are particularly important, as they integrate multiple fiber components or length scales into a unified structure. Such designs enable the synergistic combination of mechanical strength, structural stability, surface functionality, and permeability behavior. By rationally selecting fiber compositions and hierarchical structures, composite micro/nanofiber materials can be tailored to achieve high surface area, controlled porosity, and enhanced performance for advanced filtration and environmental applications.

### 4.2. Applications in Environmental Remediation

With its easily modifiable pore sizes and minimal risk of secondary contamination, filtration is a highly effective approach for mitigating environmental pollutants [67]. In the past, membranes, meshes, and adsorbents were key filtration materials for removing waterborne contaminants and controlling micro- and nanoparticle pollution. Ultrafiltration membranes, having pores smaller than microplastics, ensure complete particle retention. For instance, ultrafiltration membranes effectively prevent microplastics from passing through [68]. During the final stage of wastewater treatment, Ziajahromi et al. assessed the effectiveness of an ultrafiltration–reverse osmosis hybrid system in removing microplastics sized 100–190 μm [69]. The study demonstrated that ultrafiltration and reverse osmosis filtration reduced microplastic concentrations from 2.2 particles/L to 0.28 and 0.21 particles/L, respectively, resulting in overall removal rates greater than 90%. The MBR method treats wastewater by coupling biological processes with membrane filtration. Membranes with consistent pore structures are extensively employed, and MBR has multiple advantages relative to traditional activated sludge systems [14,70,71,72]. Illustratively, MBR has a longer solid retention time and a shorter hydraulic retention time. Despite their potential, conventional membrane-based methods for micro- and nanoplastic removal are often limited by relatively low permeability and fouling susceptibility. In contrast, micro/nanofiber membranes offer higher porosity and interconnected pore structures, enabling enhanced flux and fouling resistance. Consequently, the development of novel micro- and nanofiber-based solutions is essential.

Several approaches are available to mitigate micro- and nanoplastics, with conventional WWTPs being a widely used method. Micro/nanofiber materials, with their high surface area, customizable porosity, and functionalized surfaces, offer a particularly promising avenue for removing micro- and nanoplastics from various environmental settings [20,61,73,74]. Nanofiber membranes can efficiently remove plastic particles from water through size-based exclusion, electrostatic attraction, and hydrophobic affinity. Incorporating photocatalytic or adsorptive functionalities into the fibers enhances their ability to degrade or retain micro- and nanoplastics. Furthermore, integrating these fibers into hybrid treatment systems—such as constructed wetlands, biofilters, or membrane reactors—provides a sustainable and scalable strategy for reducing plastic pollution in aquatic environments.

**Table 1:** Examples of nanofiber-based processes and membrane performance in micro- and nanoplastic removal.

Method	Performance	Remarks	Refs.
Green photocatalytic C,N-TiO_2_ powder immobilized on a cellulose nanofiber (CNF) support	18.5% microplastic removal	After 6 h of irradiation (254 nm) A combined photocatalytic and physical approach	[75]
Polyvinylidene fluoride (PVDF) nanofiber filters	99.9% microplastic rejection	Modified with biosurfactant and metal oxides Wastewater treatment technology	[76]
Electrospun polyacrylonitrile (PAN) nanofiber membranes	100% microplastic rejection	Achieved high-flux filtration of particles as small as 0.1–0.2 μm	[77]
Electrospun polysulfone nanofibers	>99% removal of microplastics	Potential as pre-filters for particulate removal	[78]
Membrane processes for nanoplastics	Suitable	Promising for nanoplastic removal due to small pore sizes	[79]
Low-pressure driven electrospun membrane	89.9% nanoplastic removal	Efficient elimination of polystyrene (PS) nanoplastics from water	[20]
Novel metal oxide nanosheets-decorated carbon nanofibers	98% nanoplastic removal	Electrostatic interactions and metal complexation	[80]
Electrospun polyurethane (PU) nanofiber membranes	Adsorption capacity of 417 mg/g	20 wt% graphene oxide (GO)-montmorillonite (Mt)-loaded nanofibrous composite membrane (20-PGT) Micro- and nanoplastic separation	[6]
Polyethyleneimine (PEI)-modified cellulose nanofiber membrane	95.8% for nanoplastic removal	A sustainable, reusable polymer-modified cellulose nanofiber membrane Electrostatic interactions and hydrogen bonding	[81]

**Figure 4:**
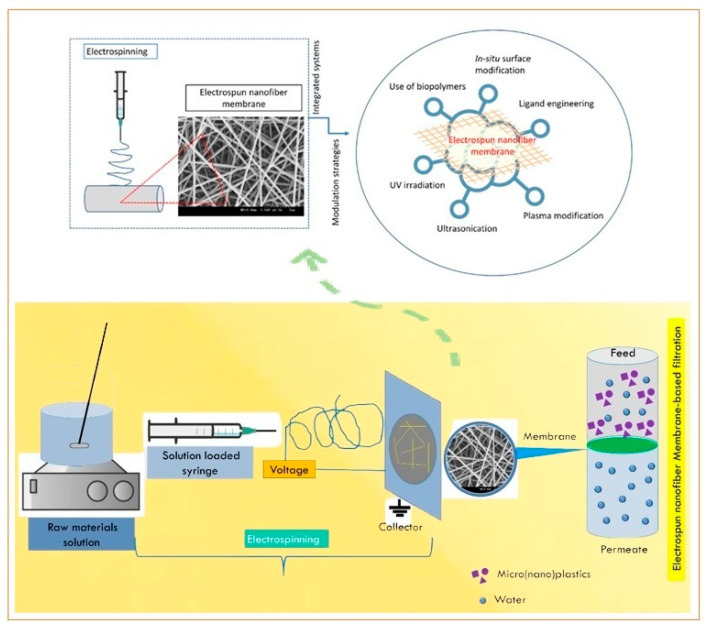
Removal of micro- and nanoplastics using electrospun nanofiber membranes [7].

Electrospun membranes, with their large specific surface area, provide extra adsorption sites; accordingly, they have been widely employed as nanofiber adsorbents to capture contaminants (Table 1). Given the effectiveness of electrospun nanofiber membranes, utilizing them to remove micro- and nanoplastics offers a promising approach for mitigating environmental pollution (Figure 4) [7]. Adjusting membrane surface properties has been identified as a potential strategy to reduce membrane fouling. Accordingly, electrospun nanofiber membrane performance can be enhanced by implementing pre-modification at the fabrication stage and post-modification on the deposited membrane surface. In a similar approach, nanofiber membranes produced by electrospinning, with adjustable morphology and porosity, have been developed as innovative microfiltration materials for highly efficient removal of particles as small as 0.1–0.2 μm from water. For example, a facile hot-pressing technique improved rejection ratios (from 0 to 100% for 0.2 μm polystyrene microplastics) in electrospun polyacrylonitrile (PAN) nanofibers [77]. These membranes delivered markedly higher flux and significantly reduced fouling while maintaining comparable particle rejection. Electrospun polysulfone nanofiber membranes showed high porosity and surface area, enabling high-flux pre-filtration with high loading capacity. The membranes exhibited a 4.6 μm bubble point and achieved >99% removal of 10–7 μm particles without permanent fouling [78]. These results highlight their strong potential as efficient filters in water treatment systems.

Recently, biofilters placed after a bioreactor demonstrate excellent microplastic removal, utilizing biofilm filtration and adsorption for both physical and biological purification [11,14]. Using cellulose nanofibers (CNF) as the matrix, 2,3-epoxypropyl trimethyl ammonium chloride (EPTMAC) as a modifier, and polyvinyl alcohol (PVA) as a crosslinker, a directionally structured CNF aerogel was fabricated through liquid nitrogen freezing and employed to adsorb small microplastics from water [82]. Modified CNF aerogels are highly promising materials for addressing plastic pollution in aquatic environments. Further, a green CNF-supported C,N-TiO_2_ photocatalyst was employed to investigate the removal of polyethylene microplastics [75]. These aerogel structures integrate CNF-based physical interception with C,N-TiO_2_ photocatalysis, leading to enhanced removal of polyethylene microplastics. Nanocellulose matrices loaded with both P25 and C,N-TiO_2_ nanoparticles exhibited increased removal efficiency across all trials. Under acidic conditions and low microplastic concentrations, CNF-C,N-TiO_2_ removed 18.5% of microplastics after 6 h of UV irradiation (254 nm), predominantly via reactive oxygen species-mediated photocatalysis. Removal by CNF occurred through photolysis, whereas CNF-P25 TiO_2_ utilized both photocatalytic and physical trapping mechanisms. The findings highlight CNF-C,N-TiO_2_ aerogels as effective agents for microplastic removal through both photocatalysis and physical capture, and stress the need to fine-tune reaction conditions to achieve optimal performance [75]. Beyond its effective performance, the aerogel structure provides notable environmental and economic benefits. In comparison, CNF are highly appealing biopolymers with extensive applications in scientific and technological fields; nonetheless, the high cost of manufacturing remains a significant barrier to the industrial-scale production of highly fibrillated, transparent CNF suspensions [83]. Embedding C,N-TiO_2_ within a CNF matrix eliminates the energy- and cost-intensive separation processes typically required for nanoparticulate photocatalysts, thereby improving feasibility for large-scale applications.

Over the past few years, advances in nanotechnology and materials science have increased the effectiveness and versatility of these materials, especially for applications that demand high selectivity and precision [20]. Properties such as pore size, surface area, and chemical characteristics control the effectiveness of these materials for various applications. Membrane systems, particularly nanofiber or electrospun types, deliver high performance and are readily scalable [61]. For example, using low-pressure driven electrospun membranes, about 90% of 50 nm nanoparticles are effectively removed from wastewater [20]. As with conventional membranes, the electrospun membrane captures suspended micro- and nanoplastics while allowing water to flow. The positively charged membrane surface enables the removal of micro- and nanoplastics of various sizes, combined with high flux and low fouling susceptibility. Due to their large surface-to-volume ratio, adjustable pore structure, and mechanical robustness, nanofiber membranes are highly effective for filtration. Yet, electrospun nanofibers, a common component, are still strongly influenced by factors including polymer selection, applied voltage, solution flow rate, and tip-to-collector distance. The characteristics of the resulting nanofibers, and thus the membrane’s filtration effectiveness, are susceptible to even minor changes in these parameters.

For environmental remediation applications, a marine biomass–derived nanofiber sponge (NF sponge/nanofiber aerogel) made from chitin was developed and utilized in an oyster-inspired filtration system. Ref. [84] highlights electrospun chitosan nanofibers for filtration. Chitosan/polyethylene oxide (PEO) nanofibers, electrospun from acetic acid using high-throughput free-surface electrospinning, achieved an average thickness of 309 ± 56 nm. Nanofiber sponges exhibit a sponge-like texture characterized by high porosity, open-cellular pores, low bulk density, and a large specific surface area. Cross-linking with glutaraldehyde improved the water stability of pristine chitosan nanofiber sponges, achieving a bulk density of 5.77 mg/cm^3^ and 99.6% porosity. Hierarchical porosity in chitosan nanofiber sponges facilitated efficient particle adsorption, as tested by polyethylene terephthalate (PET) microplastics [84]. Chitosan nanofiber sponges achieved a 99.5% reduction in NTU turbidity of the PET-microplastic suspension through hydrostatic filtration, highlighting their effectiveness in microplastic remediation.

Unlike nanofibers, polymer membranes prioritize permeability over superior filtration, which is advantageous for large-scale applications that require an optimal flow–selectivity balance [85]. Incorporating bio-based polymers can markedly enhance the sustainability of filter membranes. For instance, bio-based polyamide (PA) 6.9 shows strong potential for fabricating electrospun filter membranes (EFMs) with excellent mechanical strength and high solvent resistance. Synthesized from plant oil-derived azelaic acid, the polyamide is electrospun using a chloroform/formic acid solvent system to create self-standing electrospun nonwoven structures [86]. With porosity enabling up to 99.8% removal of PS microparticles from water, these membranes maintain their efficiency and flux over at least ten reuse cycles. This further contributes to the development of innovative materials and strategies that help mitigate plastic pollution and protect aquatic environments worldwide.

Ref. [76] emphasizes the development of advanced nanofibrous composite membranes for the efficient separation of microplastic particles (0.5 µm) and oil–water emulsions from wastewater. Polyvinylidene fluoride (PVDF) nanofibers were fabricated via a needle-free electrospinning process and laminated onto a nonwoven surface. Surface modifications using alkaline treatment, biosurfactants (BSs), TiO_2_, and CuO nanoparticles were employed to mitigate fouling and enhance separation performance, resulting in outstanding water permeability, with BS-modified membranes exceeding 9000 L/m^2^/h/bar during microplastic removal. The study provides clear evidence that membrane surface modification is an effective approach for enhancing the separation of oil–water emulsions and microplastics in wastewater treatment.

**Figure 5:**
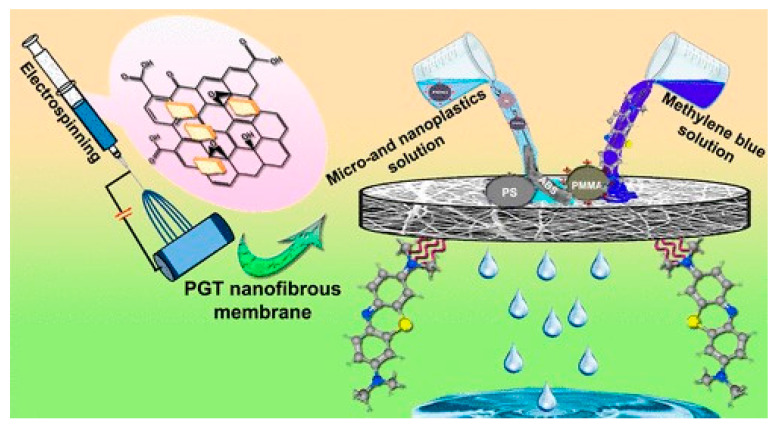
Polyurethane (PU)-based electrospun nanofiber membranes for micro- and nanoplastics separation [6].

In the context of micro- and nanoplastic removal, different methods have been employed during the synthesis of polyacrylonitrile-based electrospun nanofibers to optimize their treatment capabilities [87]. According to [6], a polyurethane (PU)-based electrospun composite membrane was developed for micro- and nanoplastics separation (Figure 5). The 20 wt% GO-Mt-loaded nanofibrous membrane (20-PGT) demonstrated superhydrophilicity (water contact angle 0°) and high water permeability. A maximum water flux of 8163 L/m^2^/h under pressure and 793 L/m^2^/h under gravity was recorded. The membrane’s performance was demonstrated using three model micro- and nanoplastics: acrylonitrile butadiene styrene (ABS), polystyrene (PS), and poly(methyl methacrylate) (PMMA) of different particle sizes. Adsorption primarily controls nanoplastic removal due to their small size, high surface area, and strong surface reactivity, which promote electrostatic, hydrophobic, and van der Waals interactions with the adsorbent. In contrast, microplastics are mainly retained through size-exclusive interception or physical sieving, as their larger dimensions allow them to be mechanically trapped within membrane pores or fibrous networks. During filtration, ABS microplastics underwent cake formation, PMMA nanoplastics experienced intermediate blocking, and PS nanoplastics were subjected to standard blocking mechanisms [6]. By combining robustness and fibrous structure, the fabricated composite membrane broadens the scope for micro- and nanoplastic removal from water.

NiFe_2_O_4_ nanosheets-decorated carbon nanofibers were synthesized by electrospinning, carbonization, hydrothermal treatment, and low-temperature calcination for nanoplastic removal [80]. SEM, FTIR, XPS, XRD, and zeta potential analyses were used to characterize the surface morphology, functional groups, chemical composition, crystal structure, and surface charge of these nanosheet-decorated nanofibers. Kinetic, isotherm, and thermodynamic studies showed that adsorption follows pseudo-second-order kinetics and the Langmuir model, suggesting chemisorption and monolayer adsorption, with a maximum capacity of 147.842 mg/g. Computational analyses demonstrate that electrostatic interactions, together with metal complexation, are the key driving forces responsible for the highly efficient removal of ultra-small polystyrene (PS) nanoplastics. The findings highlight the effectiveness of novel metal oxide nanosheet-decorated carbon nanofibers for nanoplastic removal and provide valuable guidance for the design of advanced remediation materials.

Ref. [81] demonstrated that the modified cellulose nanofiber membrane is a highly efficient nanoplastics adsorbent, showing strong potential for water treatment and environmental remediation. The modified fibers retained their original morphology, exhibited enhanced hydrophilicity and tensile strength, and achieved a high nanoplastic removal efficiency of 95.8%. Adsorption kinetic analysis followed a pseudo-second-order model, while the isotherm data were well described by the Langmuir model, indicating a maximum adsorption of 162 mg/g. Furthermore, from a sustainability perspective, the reusability of adsorbents is a key requirement for real-world applications. Deacetylated cellulose nanofiber (DCAF)–polyethyleneimine (PEI) exhibited outstanding cyclic stability, with adsorption efficiency decreasing by less than 5% after 7 reuse cycles [81]. A 100-fold scale-up experiment confirmed its practical applicability, achieving a stable removal efficiency of 92.2%, which supports its suitability for long-term and large-scale environmental remediation.

In summary, the current state of the art in micro/nanofiber-based membrane development focuses on advanced fabrication techniques, multifunctional performance, and practical scalability [21,75,88,89]. The rational design of hierarchical and multifunctional structures enables the simultaneous achievement of high permeability and precise selectivity. Increasing emphasis will be placed on sustainable and biodegradable materials, advanced surface functionalization for antifouling and self-cleaning, and integration into practical membrane modules for real-world applications.

## 5. Future Perspectives

**Figure 6:**
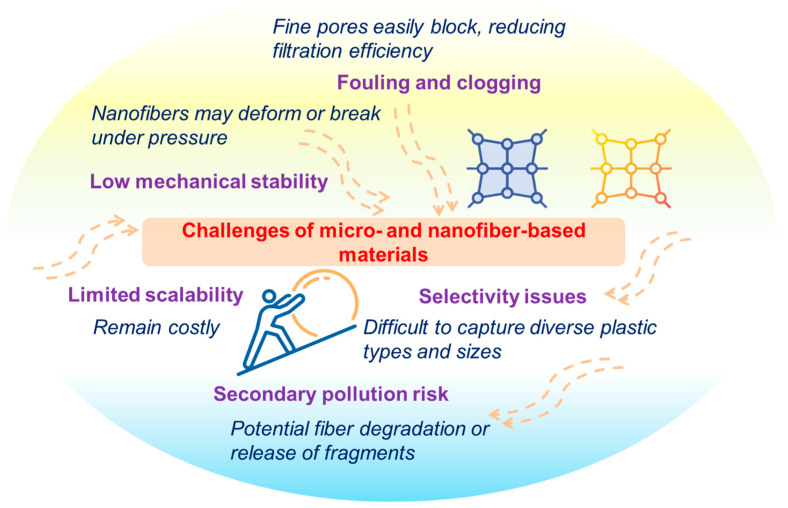
Key challenges of micro/nanofiber-based strategies for removing micro- and nanoplastics.

Overall, micro- and nanofiber-based strategies demonstrate high efficiency in removing micro- and nanoplastics due to their large surface area, tunable porosity, and strong adsorption capacity [6,20,61]. Yet, key challenges of micro/nanofiber-based strategies for removing micro- and nanoplastics include their limited long-term stability, potential fouling, and reduced efficiency in complex water matrices (Figure 6). Particular emphasis should be placed on understanding fouling mechanisms, durability over extended operation, and regeneration efficiency under realistic matrices, as these factors ultimately determine the sustainability and cost-effectiveness of large-scale implementation. In some cases, adsorption of micro- and nanoplastics by nanofiber membranes can limit permeate flow, likely due to pore clogging and cake formation, thereby reducing overall permeate output. Their performance can be further enhanced by surface functionalization and integration with photocatalytic or magnetic materials to enable selective, rapid capture of pollutants. Additionally, large-scale fabrication with consistent performance and environmentally safe disposal of used fibers remain significant obstacles. Ensuring cost-effectiveness and regeneration capability is also crucial for practical applications.

Current remediation approaches often fail to effectively address the diverse range of micro- and nanoplastics and their complex interactions across various wastewater matrices, underscoring the urgent need for highly efficient, scalable removal technologies [90]. Leveraging multifunctional capabilities—such as coupled adsorption and degradation—can significantly increase remediation efficiency. Integrating micro- and nanofiber materials with hybrid or nature-inspired treatment technologies may further support scalable, sustainable applications. Such efforts provide a critical foundation for advancing alternative materials in environmental remediation applications. For example, biopolymer-based nanofibrous materials, characterized by biodegradability, biocompatibility, sustainability, flexibility, high porosity, and tunable functionality, represent promising candidates for developing practical platforms to address environmental challenges [58]. In the future, emphasis should be placed on developing micro- and nanofiber fabrication methods that are economically viable and environmentally friendly, particularly by using biodegradable or biobased polymer materials.

Further research should increasingly focus on the rational development of advanced technical fibers and membrane systems to enhance separation efficiency and selectivity for micro- and nanoplastic removal [6,61]. One limitation is the limited consideration of micro- and nanoplastic polydispersity. The frequent use of monodisperse model particles may influence observed retention mechanisms and affect the representativeness of reported rejection efficiencies under realistic environmental conditions. Micro/nanofiber-based strategies offer unique opportunities to tailor fiber diameter, pore structure, surface charge, and chemical functionality, enabling precise control over transport and capture mechanisms across multiple size scales. Integrating multifunctional modifications, antifouling designs, and scalable fabrication approaches will be critical to advancing high-performance, durable, and technologies suitable for full-scale applications in complex environmental matrices. Also, future advances in micro/nanofiber remediation are expected to center on multifunctional and stimuli-responsive fibers that can concurrently capture, break down, and recover environmental pollutants. Progress in green synthesis, nanocomposite design, and scalable fabrication is crucial to enhancing the sustainability, selectivity, and practical implementation of micro- and nanofiber-based technologies in real-world environmental systems.

The scalability and techno-economic feasibility of micro/nanofiber-based technologies remain critical challenges for large-scale microplastic and nanoplastic remediation. Although laboratory studies demonstrate high removal efficiencies, further research is needed to optimize production costs, long-term stability, regeneration capacity, and integration into existing treatment infrastructures. Comprehensive life-cycle and cost–benefit assessments are essential to evaluate their practical applicability in real-world wastewater treatment systems.

Micro/nanofiber-based strategies, particularly those leveraging electrospun membranes and functionalized materials, offer advanced solutions for the removal and degradation of micro- and nanoplastics from water. These technologies, including nanofiber membranes and engineered materials, provide high surface area, porosity, and selectivity. However, their application requires careful management of environmental risks, such as the leaching of toxic metal catalysts and the potential for creating secondary pollution, and more environmental risks [7,91,92,93].

Globally, there is still no comprehensive strategy for tackling plastic pollution. One promising measure is the establishment of long-term policies aimed at reducing plastic waste, including improved recycling systems and the adoption of biodegradable materials. Most importantly, researchers should also examine nanosized plastics generated from the degradation of microplastics via various processes, as their small size may present substantial environmental and health risks [71,94]. A pressing challenge is developing practical techniques to remove micro- and nanoplastics from wastewater. Moving forward, efforts will focus on continuous removal at very high flow rates and low concentrations.

It could be seen that tackling global plastic pollution demands urgent, coordinated action, exemplified by efforts toward a Global Plastics Treaty. A comprehensive strategy addressing every stage of the plastic life cycle is critical. Thorough life-cycle assessments and standardized evaluation methods are crucial to ensure environmental safety and facilitate large-scale implementation. They are particularly important for assessing the sustainability of micro- and nanoplastic removal using micro- and nanoplastics. The application of nanotechnology within wastewater treatment frameworks represents a viable approach for high-efficiency, large-scale, and environmentally sustainable plastic remediation, promoting improved water quality and ecosystem integrity [25].

## 6. Conclusions

Growing concerns about pollution from excessive plastic production and improper disposal have led to intensified global efforts toward sustainability and improved waste management. Emerging micro- and nanofiber technologies have outstanding potential to combat multiple pollutants through synergistic adsorption, photocatalysis, and filtration mechanisms. Micro- and nanofiber-based materials can be applied in membrane filtration, as adsorbent fillers in batch reactors, and integrated photocatalytic units, each operating through distinct removal mechanisms. Due to their significant differences in size, surface area, colloidal stability, and transport behavior, microplastics are primarily removed through physical filtration and sieving, whereas nanoplastics often require adsorption, electrostatic interactions, aggregation, or catalytic degradation mechanisms. Membrane filtration primarily relies on size exclusion and surface interactions, offering high removal efficiency but suffering from fouling in complex water matrices. Adsorbent fillers remove nanoplastics via surface adsorption, providing tunable selectivity and low energy demand, although their performance can be hindered by coexisting contaminants in wastewater. Integrated photocatalytic units enable simultaneous capture and degradation of nanoplastics, but their efficiency is strongly influenced by water turbidity and light penetration.

As a significant outcome, recent innovations in material design, including surface modification, catalytic nanoparticle incorporation, and the utilization of biodegradable polymers, have significantly enhanced both the performance and sustainability of these materials. With their large surface area, tunable activity, and selective pollutant capture, micro- and nanofibers are emerging as strong candidates for large-scale remediation. With continued innovation and the integration of green technologies, these materials could soon transition from the laboratory to become viable environmental solutions.

Despite having several advantages, the practical efficiency of these membranes is yet to be systematically assessed under realistic operating conditions. Also, water composition plays a crucial role in determining the removal efficiency and mechanism. Parameters such as pH, ionic strength, and natural organic matter can influence particle stability, surface interactions, and aggregation behavior. Therefore, treatment performance should be evaluated under realistic water chemistry conditions rather than relying solely on results obtained in simplified laboratory media. It is required to standardize application-relevant performance metrics, such as permeability normalized by pressure and thickness, separation efficiency, and fouling resistance with cleaning recovery. In addition, mechanical stability, long-term durability, and sustainability-related indicators should be evaluated under realistic operating conditions. Achieving this goal will depend on strong collaboration among scientists, engineers, policymakers, and industry leaders to tackle the expanding challenge of micro- and nanoplastic pollution.

## Data Availability

No new data were created or analyzed in this study. Data sharing is not applicable to this article.

## Supplementary Materials

The following supporting information can be downloaded at https://www.mdpi.com/article/10.3390/membranes16070223/s1, Figure S1: Systematic Review Process Flowchart.
