# Supplementary figures and images for: From Plastic Pollution to Remediation Solutions: Micro/Nanofiber-Based Strategies for Microplastic and Nanoplastic Removal
Source: Membranes (Basel). 2026 Jun 29;16(7):223. doi: 10.3390/membranes16070223 (PMC13414061; doi:10.3390/membranes16070223)

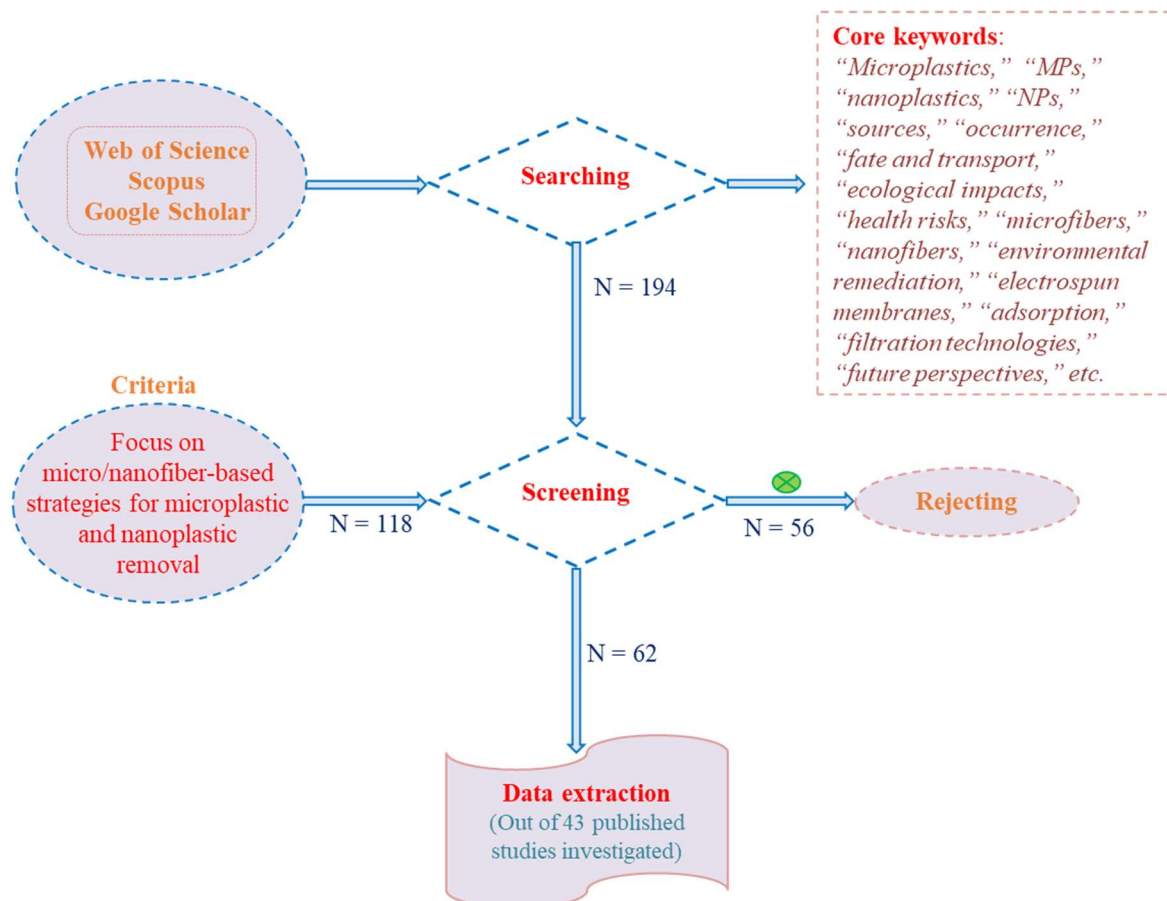

**Figure S1.** Systematic Review Process Flowchart.

Supplement: Supplementary file 1 [file membranes-16-00223-s001.zip › membranes-4147983-supplementary.pdf]
